# Development of a Polymer-Mediated Soybean Nanocomposite by Hot Melt Extrusion to Improve Its Functionality and Antioxidant Properties

**DOI:** 10.3390/foods8020041

**Published:** 2019-01-24

**Authors:** Md Obyedul Kalam Azad, Won Woo Kim, Cheng Wu Jin, Wie Soo Kang, Cheol Ho Park, Dong Ha Cho

**Affiliations:** 1 College of Biomedical Science, Kangwon National University, Chuncheon 24341, Korea; azadokalam@gmail.com (M.O.K.A.); wwkim114@gmail.com (W.W.K.); kangwiso@kangwon.ac.kr (W.S.K.); chpark@kangwon.ac.kr (C.H.P.); 2 Head of Research and Technology, Rentia Plant Factory, Chuncheon 24341, Korea; 3 College of Food Engineering, Ludong University, Yantai 264025, China; cwjin1990@gmail.com

**Keywords:** hot melt extrusion, polymer, isoflavones, phenolic content, solubility, bioaccessibility

## Abstract

The poor bioaccessibility of the phenolic compounds of soybeans is a key challenge to developing functional food products. Therefore, a novel hydrophilic food-grade hydroxypropyl methylcellulose (HPMC) polymer was added to soybean to prepare a soybean food composite (SFC), in order to improve the soybean’s functionality. The SFC was prepared with soybean (95%) plus HPMC (5%) (*w*/*w*) mixes (HSE), as well as 100% soybean extrudate (SE), at 80 °C and 130 °C by a hot melt extrusion (HME) process. A non-extrudate 100% soybean material was considered as a control. It is observed that water solubility was significantly increased (35.18%), and particle size reached to nano-size (171.5 nm) in HSE at 130 °C compared to the control (7.14% and 1166 nm, respectively). The total phenolic, flavonoid, and single isoflavones content, including daidzin, daidzein, glycitein, genistein, and genistin was significantly increased in HSE at 130 °C compared to the control. The antioxidant properties were also significantly increased in HSE at 130 °C compared to the control, measured by 2,2-diphenyl-1 picryl hydrazyl (DPPH), a ferric reducing antioxidant power assay (FRAP), and the phosphomolybdenum method (PPMD). Finally, it is concluded that the HPMC polymer could be used as a novel excipient to develop nanocomposite via HME, in order to improve the functionality of soybean food products.

## 1. Introduction

Soybean (*Glycine max* L.) is one of the healthiest legume food crops worldwide. It is a rich source of phenolic acids and flavonoids. The main flavonoids in soybean are isoflavones, a glycoside of genistin and daidzin that has a limited distribution in nature, except for soybeans [1]. Isoflavones have a high level of antioxidant capacity [2], which protects the body from various disorders, including cancer [3], cardiovascular diseases [4], and osteoporosis [5]. 

The health benefits of the bioactive compounds primarily depend on to what extent they are released from the matrix, called “bioaccessibility”, which predicts the compounds available for intestinal absorption, called “bioavailability” [6]. According to the Biopharmaceutics Classification System (BCS), isoflavones belong to class II are considered the most bioavailable but have poor bioaccessibility—for instance, the aqueous solubility and bioavailability of daidzin is 0.04 mg/mL and 40%, respectively [7,8]. Amidon et al. [9] stated that particle size reduction, an increase in amorphization, and solid dispersion are important factors to improving the bioaccessibility of class II compounds. 

**Figure 1:**
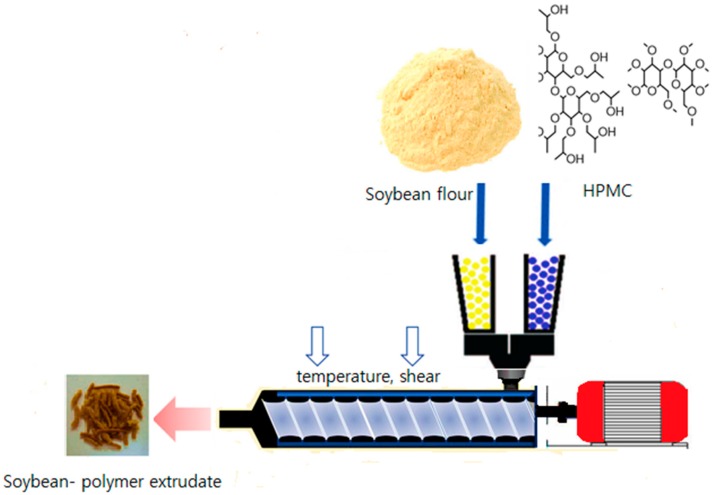
Schematic diagram of the preparation of the soybean-polymer food composite. HPMC: hydroxypropyl methylcellulose.

Many approaches have been studied to improve the solubility of the poorly water-soluble compound. Reduction of the particle size is one of the most effective ways to increase the solubility [10]. Hot melt extrusion is a novel processing technology in developing nano-size particles by the top-down technique (Figure 1). In this technique, the coarse particle is to become nano sized by high shear forces of hot melt extrusion (HME) [11]. 

The application of biopolymers is getting consideration in the food industry to improve food functionality. Hydroxypropyl methylcellulose (HPMC) is a safe food additive recommended by the European Food Safety Authority [12]. HPMC is hydrophilic and biocompatible cellulose, mainly used in the food industry as a stabilizer, emulsifier, and protective colloid [13]. It has the capabilities of hydration and gel formation, which enhance solubility, prolong the releasing time, and control the rheological characteristics of the active ingredients [14,15]. Crowley et al. [16] stated that polymers facilitate the agglomeration of compounds into granules by steric hindrance and non-covalent bonding with the polymeric chain in aqueous media. To the best of our knowledge, HPMC polymers have not been used in soybean food products yet. Therefore, in this study, HPMC is added with soybean to develop food composites by HME, in order to improve the bioaccessibility and functionality of the phenolic compounds. 

## 2. Materials and Methods

Soybean (*Glycine max* L.) flour was purchased from Chuncheon local market, Korea. Hydroxypropyl methylcellulose (HPMC) was purchased from Lotte food, Korea. Daidzin, glycitein, genistin, daidzein, glycitein, and genistein standards were purchased from Sigma (Sigma Chemical Co., St. Louis, MO, United States). Phenolic reagent (Folin Ciocalteu, 2 N), sodium bicarbonate (Na_2_CO_3_), aluminum nitrate (AlNO_3_)_3_, potassium acetate (CH_3_COO_2_K), DPPH (2,2-diphenyl-1 picryl hydrazyl), phosphate buffer, trichloroacetic acid (TCA), ferric chloride, sulfuric acid, sodium phosphate, and ammonium molybdate were purchased from Merck Chemical Corp. (Darmstadt, Germany).

### 2.1. Preparation of Soybean-Polymer Composites

The purchased coarse soybean powder was pulverized by a low-temperature turbo mill (HKP-05; Korea Energy Technology Co., Ltd., Seoul, Korea). The temperature of the mill chamber was maintained at −18 °C. The ultrafine soybean powder was stored in a desiccator until further analysis.

Soybean food composite (SFC) was prepared by hot melt extrusion (STS-25HS twin-screw HME) (Hankook E.M. Ltd., Pyoung Taek, Korea), presented in Table 1. The HME extruder was equipped with a round-shaped die (1 mm) at a feeding rate of 40 g/min at 150 rpm, with high shear of the twin screw. The processing temperature of HME was fixed at 80 °C and 130 °C. It has been reported that the processing parameter of the HME depends on the rheological characteristics of the polymer. The temperature of the HME must be higher than the glass transition temperature of a polymer to get good miscibility of the components [17]. 

### 2.2. Particle Size Analysis

The SFC (soybean extrudate (SE), soybean (95%) plus HPMC (5%) (*w*/*w*) mixes (HSE)) and control powder (0.5 g) was suspended in 50 mL of distilled water. The supernatant was separated by centrifugation at 3000 rpm for 10 min. The particle size and the polydispersity index (PI) of the supernatant was studied using a light-scattering spectrophotometer (ELS-Z1000; Otsuka Electronics, Tokyo, Japan) with three replications.

### 2.3. Solubility Measurement

One gram of SFC and the control powder was suspended in 50 mL of distilled water at room temperature. The mixture was stirred for 1 h and then centrifuged at 5000 rpm for 10 min. The supernatant was decanted into an evaporating dish of known weight. Water absorption index (WAI), water solubility (WS), and swelling power (SP) were calculated by the following formulas, described by Piao et al. [18].

$$\mathrm{Water}\;\mathrm{Absorption}\;\mathrm{Index}\;(\mathrm{WAI})=\frac{\mathrm{wet}\;\mathrm{sediment}\;\mathrm{weight}}{\mathrm{dry}\;\mathrm{sample}\;\mathrm{weight}}$$

$$\mathrm{Water}\;\mathrm{Solubility}\;(\mathrm{WS},\;\%)=\frac{\mathrm{dry}\;\mathrm{supernatant}\;\mathrm{weight}}{\mathrm{dry}\;\mathrm{sample}\;\mathrm{weight}}\times 100$$

$$\mathrm{Swelling}\;\mathrm{Power}\;(\mathrm{SP})=\frac{\mathrm{wet}\;\mathrm{sediment}\;\mathrm{weight}}{\mathrm{dry}\;\mathrm{sample}\;\mathrm{weight}\times \left(1-\frac{\mathrm{WS}\;\left(\%\right)}{100}\right)}$$

### 2.4. Extraction Protocol for Phenolic Compound Analysis

The extraction protocol was followed according to the method described by Azad et al. [19]. The SFC and control sample of 1 g was suspended in 100 mL of 80% ethanol and kept overnight in a shaker at room temperature. The extracts were filtered through Advantech 5B filter paper (Tokyo Roshi Kaisha Ltd., Saitama, Japan) and dried using a vacuum rotatory evaporator (EYLA N-1000, Tokyo, Japan) in a 40 °C water bath to obtain crude extracts. The crude extracts were freeze-dried to achieve a moisture content of <3%. Dried crude extracts were diluted using 80% ethanol to prepare 1 mg/mL stock solution and kept at −20 °C for further analysis.

### 2.5. Estimation of Total Phenolic and Flavonoid Content

Total phenolic (TP) content of SFC and control samples were determined by the Folin Ciocalteu assay [20]. In brief, a sample aliquot of 1 mL of extract (1 mg/mL) was added to a test tube containing 0.2 mL of phenol reagent (1 N). The volume was increased by adding 1.8 mL of deionized water, and the solution was vortexed and left for 3 min for reaction. Furthermore, 0.4 mL of Na_2_CO_3_ (10% in water, *v*/*v*) was added, and the final volume (4 mL) was adjusted by adding 0.6 mL of deionized water. The absorbance was measured at 725 nm after incubation for 1 h at room temperature. The TP content was calculated from a calibration curve using gallic acid, and was expressed as mg/g of gallic acid equivalent (GAE). 

The total flavonoid content (TF) content was quantified according to Ghimeray et al. [21] with slight modifications. In short, a 0.5 mL aliquot of the sample (1 mg/mL) was mixed with 0.1 mL of 10% aluminum nitrate and 0.1 mL of potassium acetate (1 M) solution. To this mixture, 3.3 mL of distilled water was added to make a total volume of 4 mL. The mixture was vortexed and incubated for 40 mins. The TF content was measured using a spectrophotometer (UV-1800 240 V, Shimadzu Corporation, Kyoto, Japan) at 415 nm. The TF content was expressed as mg/g quercetin equivalents. 

### 2.6. Analysis of a Single Isoflavone

The single isoflavone content was measured according to the modified method of Hsieh et al. [22]. The sample extract (1 mg/mL) was filtered through a 0.45 µM syringe filter (Millipore, United States) before isoflavone analysis by high-performance liquid chromatography (HPLC). An HPLC system (CBM 20A, Shimadzu Co, Ltd., Kyoto, Japan) with two gradient pump systems (LC 20AT, Shimadzu), a C18 column (Kinetex, 100 × 4.6 mm, 2.6 micron, Phenomenex), an auto sample injector (SIL-20A, Shimadzu), a UV detector (SPD-10A, Shimadzu), and a column oven (35 °C, CTO-20A, Shimadzu) were used for analysis. Solvent A was 0.4% formic acid in water, and solvent B was acetonitrile. Gradient elution was used for 0–15 min, with 33–45% B; 15–30 min, with 45–55% B; 30–40 min, with 55–80% B; and 40–45 min, with 80–33% B. The flow rate was 1.0 mL/min, the injection volume was 10 μL, and the detection wavelength was 280 nm. A standard curve was calibrated using pure standards of soybean isoflavones with high linearity (*r*^2^ > 0.995) by plotting the peak area of standard samples. The parameters like linearity, sensitivity, limit of detection (LOD), and limit of quantification (LOQ) of the HPLC method for isoflavones were validated according to the method of Montero et al. [23]. All samples were analyzed in triplicate, and the single isoflavone content was expressed as mg/g. 

### 2.7. Antioxidant Assays

#### 2.7.1. DPPH Free Radical Scavenging Capacity

The antioxidant capacity was determined on the basis of the scavenging activity of the stable 2,2-diphenyl-1 picryl hydrazyl (DPPH) free radical, according to methods described by Braca et al. [24]. The DPPH solution was prepared with 5.914 mg of DPPH powder in 100 mL of 100% methanol in the dark. Then, 1 mL of sample extract (1 mg/mL) was added to 3 mL of DPPH solution. The blank sample was prepared with 1 mL of distilled water instead of sample extract in 3 mL of DPPH solution. The mixture was shaken vigorously and left to stand at room temperature in the dark for 30 min. The absorbance was measured at 517 nm using a spectrophotometer (UV-1800 240 V, Shimadzu Corporation, Kyoto, Japan). The percent inhibition activities of the SFC and control were calculated against a blank sample using the following equation:

Inhibition (%) = [(blank sample − extract sample)/blank sample] × 100

#### 2.7.2. Ferric-Reducing Antioxidant Power Assay

The reducing power of the SFC and control samples was estimated according to the ferric reducing antioxidant power assay (FRAP) assay, as described by Pulido et al. [25] with slight modification. In brief, 1 mL of extract (1 mg/mL) was mixed with 1 mL of 0.2 M phosphate buffer, maintaining a pH of 6.6. The mixture was then incubated at 50 °C for 20 min. After incubation, 1 mL of trichloroacetic acid (TCA) was added to the solution and centrifuged at 3000 rpm for 10 min. The collected supernatant was diluted with distilled water at a 1:1 ratio. Finally, 0.25 mL of 0.1% ferric chloride was added, and the absorbance was measured at 700 nm by a spectrophotometer.

#### 2.7.3. Phosphomolybdenum Method

The total antioxidant capacity of SFC and control was assayed according to the phosphomolybdenum method (PPMD) method described by Prieto et al. [26], with slight modification. In brief, 1 mL of extract solution (1 mg/mL) was added to 3 mL of 0.6 M sulfuric acid, 28 mM sodium phosphate, and 4 mM ammonium molybdate solution. The reaction mixture was incubated at 95 °C for 150 min. The absorbance of the mixture was measured at 695 nm by spectrophotometer against a blank sample. The antioxidant capacity was expressed as the absorbance of the sample. 

## 3. Statistical Analysis

All data were expressed as mean ± standard deviation (SD) of triplicate measurements. The obtained results were compared among the soybean-polymer composite using a paired *t*-test, in order to observe the significant differences at the level of 5%. The paired *t*-test between the mean values of the SFC and control were analyzed by MINITAB (version 17.0, Minitab Inc., State College, PA, United States).

## 4. Results and Discussion

### 4.1. Analysis of Particle Size and Water Solubility

**Figure 2:**
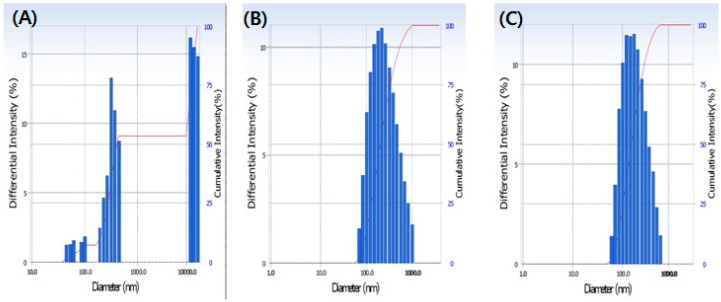
Particle size distribution of the soybean-polymer composite. (**A**) Non extrudate soybean; (**B**) extrudate soybean at 130 °C; (**C**) soybean (95%) + hydroxypropyl methylcellulose (HPMC) (5%) extrudate at 130 °C.

Particle size and distribution are important parameters of a solid formulation that estimates the rate of membrane permeability and availability of the compound in the intestine [27]. It is clearly observed from Table 2 that particle size is significantly reduced in SFC compared to the control. The nanoparticle size was recorded at 171.5 nm at 130 °C in HSE, which is significantly higher than the control (917 nm) (Table 2). Figure 2 shows the particle size distribution before and after extrusion, referred to as “polydispersity index” (PI). A splitting and wide range of particle distribution (0.52%) were observed in the non-extrudate sample, whereas narrowed particle size distribution was found in the extrudate sample (0.23%).

It is well-documented that HME is an efficient processing method to reduce particle size by a top-down technique. The high shear force generated by the twin screw of HME reduces the particle size from micro to nano [11]. Reduction of particle size is associated with transitions from the crystalline state to an amorphous state by high-energy process [28]. Enhancing bioaccessibility and bioavailability by the application of high shear is a commonly used process to obtain nanoparticles of drug compounds [29]. A decrease in particle size leads to an increase in dissolution rate through an increase in the specific surface area [30].

The PI is an important factor that affects the physical stability of nanosuspension [31]. A PI value below 0.5 indicates that the formulation is homogeneous [32]. All PI values of all samples, except the control, were below 0.4, thus showing that all samples formed homogeneous nanosuspensions. 

The water absorption ability of the SFC and the control were measured by the parameter of the water solubility (WS), water absorption index (WAI), and swelling power (SP), as shown in Table 3. It is noted that the highest WS (35.18%) was achieved in HSE at 130 °C, as compared to the control (7.14%). The SP was reduced in polymer-mediated formulation compared to the control; however, there was no significant difference observed for WAI among the formulations. 

Aqueous solubility is an important limiting factor for the bioavailability of the BCS class II compounds. Water solubility and swelling power provide evidence of interactions between the water molecules and the starch chains in the crystalline and amorphous regions of a matrix. The water absorption index represents the ability of a substance to associate with water under a limited water condition.

Miller et al. [33] investigated the impact of HPMC polymers on the ability to improve the solubility and bioavailability of the active drug compound process by HME. They stated that HPMC improves solubility due to its ability to provide hydrogen bonding donor sites. The formation of hydrogen bonding prevents migration of the active molecules within the polymer matrix. Zheng et al. [34] explored solubility improvement of poorly water-soluble active molecules, by producing solid dispersions with HPMC processed by HME. The efficacy of the HPMC to enhance the solubility of the poorly soluble compound is largely due to its ability to prevent recrystallization of the molecules in aqueous media [35,36]. The swelling behavior of the extrudate soybean composite tends to be reduced, which may be due to the hydrolysis of starch in the extrudate soybean-polymer composite. Tester and Morrison [37] stated that the swelling behavior of cereal is reduced due to amylose, which acts as an inhibitor of swelling. The WAI and SP were found to be reduced in corn, which is thought to be due to the collapse of the fibrous structures by a high shearing force of the twin screw [38].

### 4.2. Analysis of Bioactive Compounds and Antioxidant Capacity

**Figure 3:**
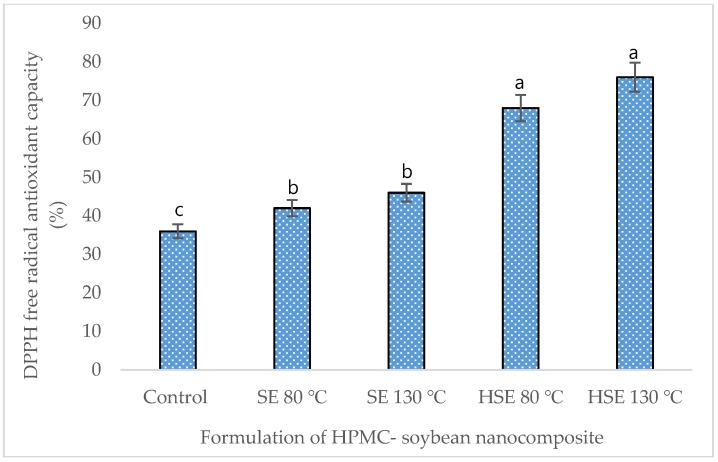
DPPH (2,2-diphenyl-1 picryl hydrazyl) free radical scavenging activity of the soybean composite. Mean values ± SD from triplicate separated experiments are shown. Control: non-extrudate soybean, SE: extrudate 100% soybean at a different temperature, HSE: soybean (95%) + HPMC (5%) extrudate at a different temperature.

**Figure 4:**
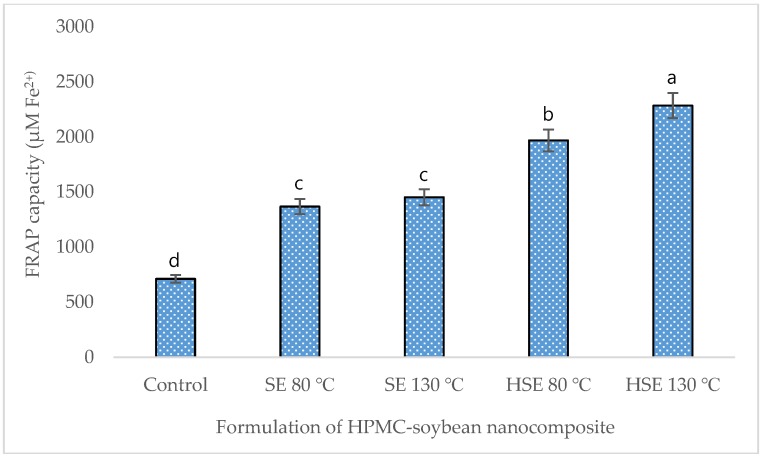
Reducing power of soybean-polymer composite (ferric reducing antioxidant power assay (FRAP assay)). Mean values ± SD from triplicate separated experiments are shown. Control: non-extrudate soybean, SE: extrudate 100% soybean at a different temperature, HSE: soybean (95%) + HPMC (5%) extrudate at a different temperature.

**Figure 5:**
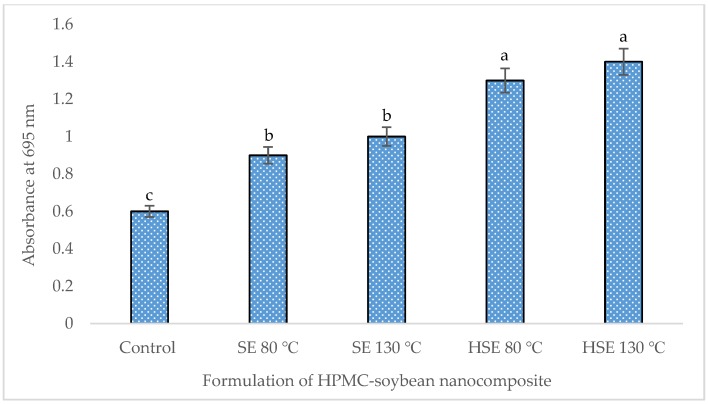
Total antioxidant capacity of soybean-polymer composite (phosphomolybdenum method (PPMD)). Mean values ± SD from triplicate separated experiments are shown. Control: non-extrudate soybean, SE: extrudate 100% soybean at a different temperature, HSE: soybean (95%) + HPMC (5%) extrudate at a different temperature.

In this study, the highest extraction of total phenolic (9.82 mg/g) and flavonoid content (4.27 mg/g) was achieved in HSE at 130 °C, compared to the control (5.40 mg/g, 1.82 mg/g, respectively) (Table 4). Glycosides and aglycones of soybean isoflavones, including daidzin, daidzein, glycitein, genistein, and genistin were extracted by 1.70, 0.82, 0.58, 1.02, and 1.84 mg/g, respectively, in HSE at 130 °C, which is significantly higher than the control (Table 5). The total antioxidant capacity of the extrudate formulation was higher in HSE at 130 °C, measured by DPPH, FRAP, and PPMD (Figure 3, Figure 4 and Figure 5).

The increase of the phenolic and flavonoid may be owing to the increase in the extractability of bound phenolics by the thermal degradation of the cellular constitutes [14]. Youna et al. [39] showed that reduced bran particle size by mechanical treatment resulted in increased bioaccessibility of phenolic acid. The increase of the phenolic profile in other food materials during extrusion processing has also been reported by another researcher [40,41]. Mahungu et al. [42] observed that acetyl derivatives of genistin and daidzin are increased with increasing extrusion temperature. High temperature cause malonyl conjugates to undergo heat-induced decarboxylation and desertification of isoflavone derivatives. 

The increased antioxidant property after processing might be because of the breakdown of cellular constituents and membranes of the materials. The formation of a variety of intermediate byproducts during processing might contribute to antioxidant properties [43]. Denaturation of proteins during extrusion leads to open loose structures, which promote tannin-protein interaction and form tannin-protein complexes that enhance antioxidant capacity [44]. White et al. [45] observed that antioxidant activity is increased in extrudate products with elevated barrel temperature.

The bioaccessibility of the phenolic compound from soybean-polymer composites was increased in 130 °C. In general, the processing temperature in the HME extruder needs to be above the glass transition temperature of a given polymer to embed the active compound into a polymeric matrix [45]. In this study, the increased bioaccessibility of phenolic compounds verified good miscibility between the soybean compound and HPMC polymer at 130 °C compared to 80 °C. Riaz et al. [46] reported that temperature-sensitive phenolic compounds may undergo decarboxylation during extrusion, which reduces their bioaccessibility and antioxidant capacity. However, the addition of a polymer extends the thermal stability of active compounds in a form of the solid formulation. HPMC has proven to be an excipient for providing thermal stability to the low thermal stable compound during processing [47].

## 5. Conclusions

HPMC polymers significantly increased the functional quality of melt extrudate soybean. It was observed that nano-size particles, higher solubility, and increased phenolic content and antioxidant capacity were achieved in a polymer-mediated soybean composite when processed at 130 °C. Therefore, it would be concluded that HPMC polymers could potentially be used in order to improve the bioaccessibility of bioactive compounds of soybean processed by HME.

## Figures and Tables

**Table 1:** The soybean-polymer compositions and hot melt extrusion (HME) conditions of developed formulations.

SFC Materials	Mixing Ratio (*w*/*w*) (%)	HME Condition	HME Temperature (°C)	Formulation
Soybean	100	Non extrusion	-	Control
Soybean	100	Extrusion	80	SE
Soybean	100	Extrusion	130	SE
Soybean-HPMC	95-5	Extrusion	80	HSE
Soybean-HPMC	95-5	Extrusion	130	HSE

SFC: soybean food composite; HPMC: hydroxypropyl methylcellulose; HME: hot melt extrusion; SE: solid extrudate; HSE: HPMC mediated solid extrudate.

**Table 2:** Particle size analysis of the supernatant of the suspension for the soybean-polymer composite.

Formulation	Particle Size (nm)	Polydispersity Index (%)
^a^ Control	917.0 ± 54.0a	0.52 ± 0.13a
^b^ SE 80 °C	260.5 ± 6.9b	0.28 ± 0.01b
^b^ SE 130 °C	252.5 ± 7.3b	0.26 ± 0.01b
^c^ HSE 80 °C	197.1 ± 6.6c	0.31 ± 0.01b
^c^ HSE 130 °C	171.5 ± 11.6c	0.23 ± 0.02b

Mean values ± SD from triplicate separated experiments are shown. Value marked by different letters in each column is significantly different by *t*-test (*p* < 0.05). ^a^ Non-extrudate soybean; ^b^ extrudate 100% soybean at different temperature; ^c^ soybean (95%) + hydroxypropyl methylcellulose (HPMC) (5%) extrudate at different temperatures.

**Table 3:** Water absorption-related parameters of the soybean-polymer composite.

Formulation	Water Solubility (%)	Water Absorption	Swelling Power
^a^ Control	7.14 ± 0.92d	3.33 ± 0.42a	5.35 ± 0.31a
^b^ SE 80 °C	17.64 ± 0.31c	3.52 ± 0.04a	3.24 ± 0.24a
^b^ SE 130 °C	22.75 ± 1.62b	3.27 ± 0.92a	3.36 ± 0.15a
^c^ HSE 80 °C	25.51 ± 1.84b	3.70 ± 0.52a	2.84 ± 0.17b
^c^ HSE 130 °C	35.18 ± 2.50a	3.17 ± 0.44a	2.48 ± 0.36b

Mean values ± SD from triplicate separated experiments are shown. Value marked by different letters in each column is significantly different by *t*-test (*p* < 0.05). ^a^ Non-extrudate soybean; ^b^ extrudate of 100% soybean at a different temperature; ^c^ soybean (95%) + HPMC (5%) extrudate at different temperature.

**Table 4:** The total phenolic and flavonoid content analysis from soybean-polymer food composites.

Formulation	Total Phenolic Content (mg/g)	Total Flavonoid Content (mg/g)
^a^ Control	5.40 ± 0.11c	1.82 ± 0.68c
^b^ SE 80 °C	6.52 ± 0.96b	2.24 ± 0.47b
^b^ SE 130 °C	6.60 ± 1.28b	2.98 ± 0.29b
^c^ HSE 80 °C	8.71 ± 0.88a	3.29 ± 1.1a
^c^ HSE 130 °C	9.82 ± 0.21a	4.27 ± 1.2a

Mean values ± SD from triplicate separated experiments are shown. Value marked by different letters in each column are significantly different by *t*-test (*p* < 0.05), ^a^ non-extrudate soybean, ^b^ extrudate of 100% soybean at a different temperature, ^c^ soybean (95%) + HPMC (5%) extrudate at a different temperature.

**Table 5:** Analysis of single isoflavone content from the soybean-polymer food composite (mg/g).

Formulation	Daidzin	Glycitin	Genistin	Daidzein	Glycitein	Genistein
^a^ Control	0.13 ± 0.01c	N.D.	N.D.	0.19 ± 0.04c	0.30 ± 0.02b	0.43 ± 0.03c
^b^ SE 80 °C	0.21 ± 0.05b	0.51 ± 0.04a	1.04 ± 0.08b	0.21 ± 0.06c	0.27 ± 0.05b	0.69 ± 0.01b
^b^ SE 130 °C	0.28 ± 0.07b	0.48 ± 0.21a	0.92 ± 0.11b	0.35 ± 0.11b	0.34 ± 0.09a	0.95 ± 0.27a
^c^ HSE 80 °C	1.46 ± 0.04a	0.55 ± 0.02a	1.67 ± 0.05a	0.59 ± 0.02b	0.32 ± 0.02a	0.83 ± 0.24a
^c^ HSE 130 °C	1.70 ± 0.02a	0.58 ± 0.04a	1.84 ± 0.03a	0.82 ± 0.03a	0.46 ± 0.05a	1.02 ± 0.06a

Value marked by different letters in each column are significantly different by *t*-test (*p* < 0.05), ^a^ non-extrudate soybean, ^b^ extrudate of 100% soybean at a different temperature, ^c^ soybean (95%) + HPMC (5%) extrudate at a different temperature N.D.: not detected.
